# Construction of a prediction model for axillary lymph node metastasis in breast cancer patients based on a multimodal fusion strategy of ultrasound and pathological images

**DOI:** 10.3389/fonc.2025.1591858

**Published:** 2025-09-09

**Authors:** Lingli Peng, Lan Yu, Beibei Liu, Feixiang Xiang, Yu Wu

**Affiliations:** ^1^ Department of Ultrasound, Union Hospital, Tongji Medical College, Huazhong University of Science and Technology, Wuhan, China; ^2^ Department of Pathology, Union Hospital, Tongji Medical College, Huazhong University of Science and Technology, Wuhan, China

**Keywords:** breast cancer, deep learning, axillary lymph node metastasis, ultrasound, core-needle biopsy

## Abstract

**Background:**

Accurate assessment of axillary lymph node status is essential for the management of breast cancer. Recent advancements in deep learning (DL) have shown promising results in medical image analysis. This study aims to develop a multimodal DL model that integrates preoperative ultrasound images and hematoxylin and eosin (H&E)-stained core needle biopsy pathology images of primary breast cancer to predict axillary lymph node metastasis (ALNM).

**Materials and methods:**

This study included 211 patients with histologically confirmed breast cancer, conducted between February 2023 and March 2024. For each patient, one ultrasound image and one histopathological image of the primary breast cancer lesion were collected. Various DL architectures were applied to extract tumor features from the ultrasound and histopathology images, respectively. Multiple fusion strategies, combining features from both ultrasound and pathology images, were developed to enhance the comprehensiveness and accuracy of predictions. The performance of the single-modality models, multi-modality models, and different fusion strategies were compared. Evaluation metrics included precision, accuracy, recall, F1-score, and area under the curve (AUC).

**Results:**

PLNeT and ULNet were identified as the most effective feature extractors for histopathological and ultrasound image analysis, respectively. Overall, the multilayer fusion model outperformed single-modality models in predicting ALNM, achieving an accuracy of 0.7353, precision of 0.7344, recall of 0.7576, F1-score of 0.7463, and AUC of 0.7019.

**Conclusion:**

Our study provides a multilayer fusion strategy using ultrasound and pathology images of the primary tumor to predict ALNM in breast cancer patients. Although achieving suboptimal performance, this model has the potential to determine appropriate axillary treatment options for patients with breast cancer.

## 1 Introduction

Breast cancer (BC) is the most common malignant tumor among women globally and has become the second leading cause of cancer-related mortality in this population, with a reported mortality of approximately 15% (1). Axillary lymph node metastasis (ALNM) is one of the most significant predictors of overall recurrence and survival in breast cancer patients (2), with each positive lymph node associated with an increased risk of death by approximately 6% (3). Accurately assessing whether axillary lymph nodes are metastatic is a critical step in the management of breast cancer (4, 5). Misdiagnosis of axillary lymph nodes can lead to missed surgical opportunities and may increase unnecessary surgical trauma and complications (6). Consequently, precise prediction of lymph node status in breast cancer patients is essential for effective management of surgical strategies.

Currently, the assessment of axillary lymph nodes primarily relies on imaging studies (such as ultrasound and MRI) and sentinel lymph node biopsy (SLNB) (7, 8). While imaging can provide valuable insights regarding metastasis, its accuracy is typically limited by operator experience, ranging from 70% to 85%, which can lead to misdiagnosis and delayed treatment (9). SLNB, as a minimally invasive procedure, can enhance accuracy to over 90%, but it may also overlook metastases in non-sentinel lymph nodes (6). Additionally, there are potential side effects, including lymphedema and upper limb numbness (10). Therefore, there is an urgent need to identify a reliable and effective alternative method for accurately assessing the axillary lymph node status in breast cancer patients, facilitating informed decisions regarding axillary management.

Radiomics, through high-throughput extraction of numerous features from medical images, has been applied in the prediction of ALNM in breast cancer patients (11–13). Some studies have constructed deep learning (DL) models based on clinicopathological data, ultrasound or MRI images, aimed at improving the detection accuracy of ALNM, with the area under the curve (AUC) ranging from 0.74 to 0.89 (14–16). However, as single-modal models, these methods have certain limitations, including information constraints (which may not comprehensively capture the biological characteristics of the lesions), lack of diversity (where different patients may exhibit similar imaging features despite significant differences in their pathological status), and insufficient model validation (with a lack of external validation data potentially affecting the generalizability of the results). With the rapid development of deep learning technology, pathological information has also gradually been integrated into high-throughput analysis. Xu et al. studied a cohort of early breast cancer patients who underwent preoperative core needle biopsy (CNB) and found that DL models based on primary tumor biopsy slices could effectively refine the prediction of ALNM, which achieved an AUC of 0.816 (17). This finding underscores the importance of pathological slices in predicting ALNM. Multimodal fusion model contains more comprehensive information than single-modal models. For instance, Bove et al. developed a model that combined clinical and radiomic features provided the best performances to predict the nodal status in clinically negative breast cancer patients, achieving an AUC value of 0.886 (18). If radiomics, which focuses on macro-level imaging features, can be combined with histopathology, which addresses micro-level characteristics, this multimodal approach will facilitate the integration of diverse information, thereby further enhancing the accuracy of predictions.

Therefore, we hypothesize that the combination of breast ultrasound and puncture pathology analysis may yield encouraging results in preoperatively distinguishing and predicting ALNM. In this research, we seek to establish a predictive model for ALNM in breast cancer using a multimodal fusion strategy, incorporating pre-treatment ultrasound images and hematoxylin and eosin (H&E)-stained core needle biopsy pathology of primary lesion.

## 2 Materials and methods

### 2.1 Patients

A retrospective collection of imaging and clinical data was conducted for patients diagnosed with breast cancer via CNB at Union Hospital of Tongji Medical College of Huazhong University of Science and Technology between February 2023 and March 2024. The pathological diagnosis methods for axillary lymph nodes included ultrasound-guided CNB, sentinel lymph node biopsy (SLNB), and axillary lymph node dissection (SLND). The inclusion criteria were: (1) availability of preoperative ultrasound images of the breast and axillary lymph nodes from our hospital, with complete imaging; (2) ultrasound-guided CNB performed following breast ultrasound examination; (3) breast surgery conducted 1–2 weeks after the biopsy. The exclusion criteria were: (1) patients with a history of contralateral breast cancer or who had undergone preoperative chemotherapy or radiotherapy; (2) patients with bilateral breast cancer or multiple lesions that made target delineation difficult; (3) tumors that were excessively large, exceeding the measurable range of the ultrasound probe. For each patient, one ultrasound image and one histopathological image of the primary breast cancer lesion were obtained.

This study was approved by the Ethics Committee of Union Hospital at Huazhong University of Science and Technology, and the methods were applied in accordance with the approved guidelines. Informed consent was obtained from all patients.

### 2.2 Data collection

#### 2.2.1 Ultrasound examination

Breast ultrasound examinations were performed by one of ten experienced radiologists following standard practice protocols. Several ultrasound devices from manufacturers such as GE Healthcare, Mindray, and Philips were equipped with linear transducers (frequency range of 10–18 MHz) to capture breast and ultrasound images. Unlabeled two-dimensional images stored in the maximum longitudinal plane of the breast lesions were utilized for subsequent analysis. Additionally, axillary ultrasound reports for each patient were recorded and categorized as either suspicious or unsuspicious. Suspicious sonographic features of axillary lymph node metastasis (ALNM) included irregular morphology, absence of the fatty hilum, eccentric cortical thickening (>3 mm), and microcalcifications (7, 8).

#### 2.2.2 Biopsy and pathological image collection

Core biopsy samples were obtained using a 16-gauge hollow needle under ultrasound guidance. Each lesion was punctured two to three times, with harvested tissue immediately fixed in 10% neutral buffered formalin for 6–72 hours. Surgical specimens were directly sampled from cancerous lesions and fixed for 24–72 hours. All tissues were subsequently embedded in paraffin, sectioned at 5 μm intervals, and stained with hematoxylin and eosin (H&E). Following the methodology described by Yang et al. for oral squamous cell carcinoma analysis (19), two senior breast pathologists evaluated the H&E slides and selected representative tumor regions for microscopic imaging independently. Image acquisition was performed at 10× objective (NA=0.30) and 10× eyepiece magnification using Olympus BX53 and Nikon Eclipse Ni microscopes equipped with DP27 digital cameras (2048 × 1536 pixel resolution). Spatial resolution was calibrated to 1.25 μm per pixel using a NIST-traceable stage micrometer, with each image covering a 2.0 × 1.5 mm tissue region. Representative region selection followed established pathological protocols requiring ≥30% tumor cellularity and avoidance of necrosis/artifact zones.

#### 2.2.3 Clinicopathological characteristics

Clinical data recorded included patient age, menopausal status and tumor location. Histological type and immunohistochemical results for estrogen receptor (ER) status, progesterone receptor (PR) status, human epidermal growth factor receptor 2 (HER2) status, and Ki-67 levels were also documented based on the entire tumor surgical specimen. Ki-67 positive was defined as proliferation index at least 14% (20). All BC patients were categorized into three molecular subtypes according to their immunohistochemical results: Luminal (ER and/or PR-positive), HER2 overexpression (ER and PR-negative, HER2-positive) and triple-negative breast carcinoma (TNBC, ER, PR, and HER2-negative).

### 2.3 Image data processing

#### 2.3.1 Manual annotation of ROI

This study required further processing of paired ultrasound images for each lesion to ensure the model focused on critical feature areas. Specifically, one ultrasound physician with over 10 years of clinical experience manually annotated the regions of interest (ROIs) in the ultrasound images. Utilizing their extensive clinical expertise, this physician accurately identified and localized the tumor areas, subsequently drawing the smallest bounding rectangles that encompassed the tumor regions. Another ultrasound physician, also with over 10 years of clinical experience, was responsible for the review process. This manual annotation procedure strictly adhered to a standardized annotation protocol to ensure consistency and accuracy in the annotations.

#### 2.3.2 Data preprocessing

The data preprocessing in this study involved the processing of pathological images and ultrasound data, with the aim of providing standardized data input for subsequent model training. Pathological images underwent data cleaning and preprocessing. All pathological images were uniformly resized to 224x224 pixels to meet the input requirements of the deep learning model. Data augmentation techniques were employed to increase image diversity, primarily involving random resizing and cropping (RandomResizedCrop) and random horizontal flipping (RandomHorizontalFlip) to simulate different angles and positions of capture. Additionally, the image data were standardized to a distribution with a mean of 0.5 and a standard deviation of 0.5 to enhance the stability and generalization capability of model training.

In the preprocessing of ultrasound images, a similar pipeline data augmentation was followed, including resizing all images to 224×224 pixels and applying random cropping and horizontal flipping for data augmentation. Vertical flipping was intentionally avoided due to anatomical orientation constraints. To address the inherent heterogeneity caused by the use of ultrasound scanners from different manufacturers—namely GE Healthcare, Mindray, and Philips—we implemented additional harmonization techniques aimed at reducing inter-scanner variability. Specifically, we applied z-score normalization to standardize the intensity distribution of each image, followed by histogram equalization to align contrast and grayscale across different devices. These procedures were intended to minimize variations stemming from differences in hardware resolution and signal processing. Additionally, mask-guided annotation was utilized to highlight tumor-specific regions, enabling the model to concentrate on lesion-relevant features while reducing the influence of peripheral artifacts and device-specific noise. By combining normalization, histogram alignment, and spatial focus through masking, the ultrasound images were standardized to better support consistent feature learning across multi-source inputs.

### 2.4 Construction of deep learning model

This study established a multimodal deep learning model to perform feature extraction and fusion on ultrasound imaging data and pathological image data, aiming to predict the likelihood of axillary lymph node metastasis in breast cancer.

#### 2.4.1 Construction of imaging model

The imaging model utilized classic convolutional neural network (CNN) architectures, ResNet50 and DenseNet, to extract tumor features from ultrasound images. After data preprocessing, the input ultrasound images were first processed through a series of convolutional and pooling layers to extract embedding representations containing high-level features. To prevent model overfitting, a Dropout layer (with a value set to 0.5) was applied before the fully connected layer of the network. During the model training process, to accelerate convergence and improve accuracy, transfer learning was employed using pretrained weights from ResNet50 and DenseNet models. The learning rate was set to 0.001 and gradually decayed by half every 10 epochs. Model parameters were optimized using the Adam optimizer, chosen for its adaptive learning rate adjustment and stable convergence, especially when training from pretrained backbones with moderate data sizes. We designate the name ULNet to represent the best-performing standard networks for ultrasound image analysis. ULNet corresponds to a modified ResNet50 architecture with strategic adaptations for ultrasound images analysis, selected due to its strong performance in image-based classification tasks and proven ability to capture spatial and edge-related features—especially suitable for ultrasound’s texture and boundary information.

#### 2.4.2 Construction of pathological model

Due to the high-resolution features of pathological images, more detailed feature extraction methods are required. In this study, the Vision Transformer (ViT) and PLNet models were selected, as they are suitable for processing high-resolution images. ViT segments the pathological images into 16x16 image patches and feeds these patches into multiple layers of Transformer encoders to obtain a more global feature representation. It should be noted that PLNet is not a novel architecture, but rather a naming convention adopted to represent the best-performing standard networks for pathological images. PLNet is a lightweight CNN architecture optimized for pathology images, featuring four convolutional blocks with progressive filter expansion and global pooling. It was selected over more complex architectures like ViT due to its better balance between global context and convergence stability on high-resolution image data. The input pathological images were normalized and resized to 224x224 pixels, and features were extracted using both the ViT and PLNet models, resulting in embeddings that represent the pathological images. During training, the learning rate for the ViT model was set at 3e-5, while for PLNet it was set at 1e-4, with both utilizing the Adam optimizer. Batch Normalization layers were incorporated during feature extraction to eliminate channel discrepancies and enhance the model’s robustness on pathological images.

#### 2.4.3 The strengths and weaknesses of ULNet and PLNet models

In terms of strengths, ULNet offers deep feature hierarchies and residual learning that mitigate vanishing gradients, while PLNet excels in efficient representation learning with fewer parameters, particularly helpful under limited data conditions. A noted limitation is that ULNet may be sensitive to image artifacts or probe differences, and PLNet, as a simpler CNN, may underperform on highly heterogeneous pathology data unless supported by proper normalization and augmentation.

#### 2.4.4 Construction of multimodal fusion model

The multimodal fusion model combines features from both ultrasound images and pathological images to enhance the comprehensiveness and accuracy of model predictions. Initially, feature embeddings are extracted separately through the imaging and pathological models, followed by the fusion of information from the two modalities at the feature level. Various fusion strategies were explored, including concatenation, weighted fusion, attention mechanisms, and multilayer fusion. In the multilayer fusion strategy, the fusion module operates as follows: First, each modality-specific feature vector is individually processed through batch normalization layers to reduce scale discrepancies. The normalized features are then concatenated into a single vector. This combined vector is passed through a fully connected (dense) layer with 256 neurons, followed by ReLU activation. A subsequent Batch normalization layer and Squeeze-and-Excitation (SE) module are applied to reweight channel-wise dependencies and enhance feature importance adaptively. The output is then fed into a final classification head consisting of a single neuron with sigmoid activation for binary classification. All activation functions used are standard ReLU (except for the final sigmoid), and dropout was not applied within the fusion module to retain feature integrity. The learning rate for the multimodal fusion model was set at 1e-4, utilizing the AdamW optimizer, with the learning rate decayed every 5 epochs to stabilize the fusion effect.

**Figure 1:**
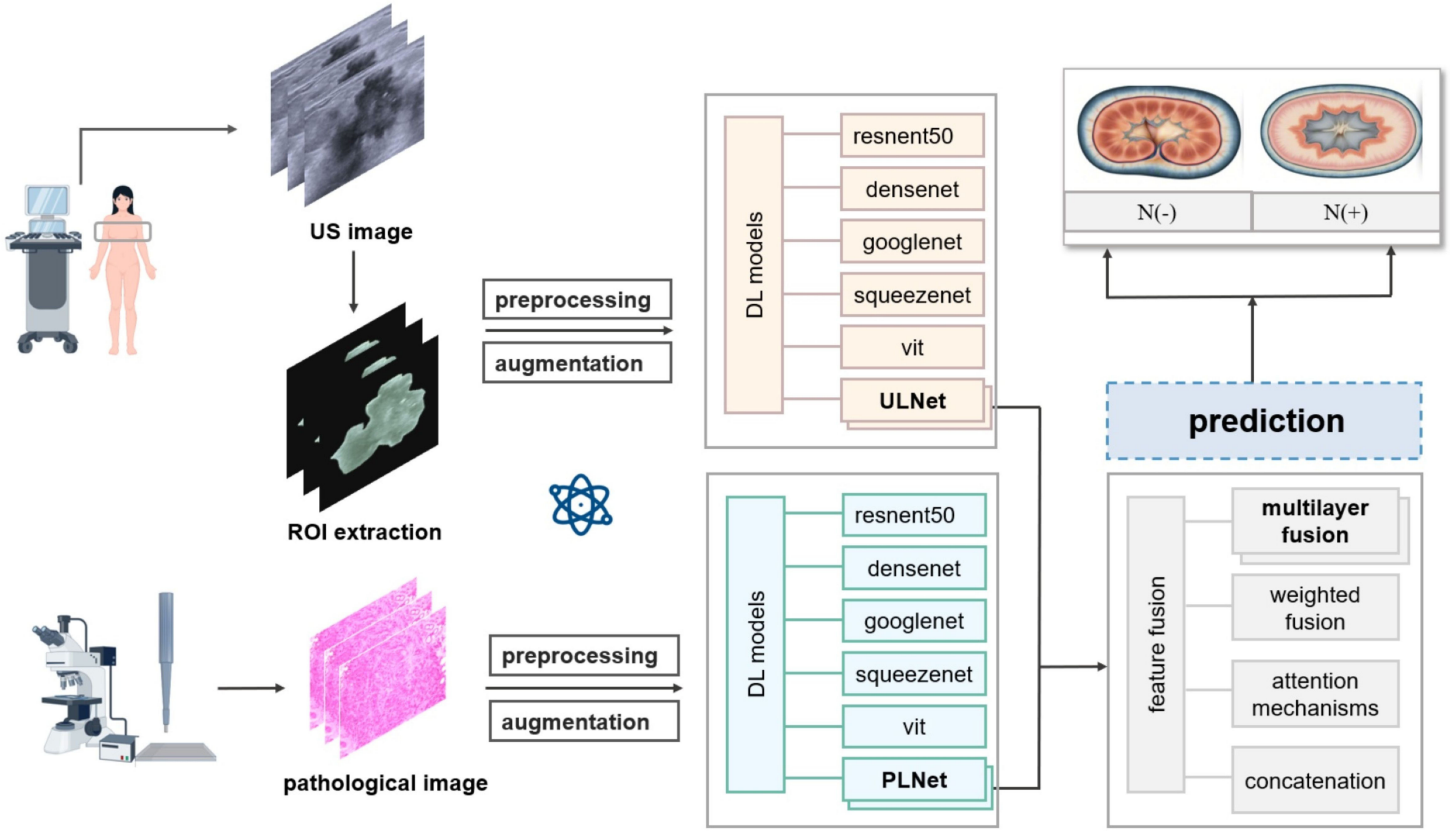
Flowchart of deep learning model to predictive axillary lymph node metastasis in breast cancer.

For the multimodal fusion model, we employed AdamW, an improved variant that decouples weight decay from gradient updates. This choice was based on the empirical observation that AdamW offers more robust generalization in joint optimization settings, where fused features from two distinct modalities may introduce higher gradient variance and overfitting risks. AdamW’s stronger regularization capability helped stabilize fusion training and improve convergence without extensive fine-tuning. The detailed DL flowchart is illustrated in **Figure 1**.

### 2.5 Experimental setup

In the experimental setup of this study, to ensure an effective evaluation of model performance, the dataset was randomly divided into training and testing sets at a ratio of 8:2. The model training utilized the AdamW optimizer, and hyperparameter tuning was performed through grid search, including parameters such as learning rate and batch size, to ensure optimal model performance. During training, the initial learning rate was set to 0.001, with a reduction by half every 5 epochs to accommodate the convergence requirements of the model. Model training and validation were conducted on an NVIDIA RTX 3090 GPU cluster, with each GPU equipped with 24GB of memory, utilizing a total of 5 GPUs, and the PyTorch deep learning framework for model construction and training. The programming language used was primarily Python 3.8, with the experimental environment comprising the Ubuntu 20.04 operating system, supporting CUDA 11.1 to accelerate GPU computations. Key Python libraries required for the experiments included torch, torchvision, numpy, pandas, and scikit-learn. Additionally, to ensure the reproducibility of the experiments, the random seed was uniformly set during both data splitting and model training processes.

### 2.6 Model evaluation

For model evaluation, we employed multiple metrics to comprehensively assess model performance, including accuracy, precision, recall, and F1-score, to evaluate the classification effectiveness of the model in predicting axillary lymph node metastasis. For the binary classification task (metastatic *vs*. non-metastatic), a confusion matrix was used to analyze the model’s classification capabilities and misclassification instances. Furthermore, to further validate the model’s discriminative ability, we plotted the ROC curve and calculated the AUC value, with an AUC value closer to 1 indicating better discriminative performance. To assess the enhancement effect of multimodal fusion on the model, we compared the performance differences between the unimodal (imaging or pathological) models and the fusion model, focusing on improvements in recall and accuracy.

Statistical analysis of experimental results was conducted using SPSS software (V.27.0). Continuous variables were compared using either a student’s t-test or the Mann-Whitney U test, depending on the normality of the distribution. These variables are presented as mean ± standard deviation (SD) or median (interquartile range), as appropriate. The chi-square test was used to assess differences between categorical variables, which are presented as frequencies and percentages. A p-value of < 0.05 was considered statistically significant.

## 3 Results

### 3.1 Clinicopathological characteristics of patients

This study enrolled a total of 211 female BC patients between February 2023 and March 2024, with a mean age of 53.1 ± 11.7 years (ranging from 23 to 85 years). All participants underwent axillary lymph node biopsy and/or dissection, which identified ALNM in 107 patients, while the remaining 104 cases were node-negative. Patients were categorized into two groups according to axillary lymph node status. **Table 1** summarizes the demographic and clinicopathological characteristics of the study population. Univariate analysis showed no significant differences between the two groups in terms of age, menopausal status, tumor location, histological types, hormone receptor status, or molecular subtypes (P > 0.05). However, significant differences were observed in the Ki-67 index, HER2 status, and preoperative axillary ultrasound findings (P < 0.05). Axillary ultrasound demonstrated a true positive rate of 52.3% (56/107), a false negative rate of 47.7% (51/107), a true negative rate of 90.4% (94/104), and a false positive rate of 9.6% (10/104).

**Table 1:** The demographic and clinicopathological characteristics of breast cancer patients.

Characteristics	ALN (+) N=107	ALN (–) N=104	P value
Mean age (y)	52.9 ± 11.3	53.4 ± 12.2	0.55
Menopause status (%)			0.92
premenopausal	61 (57%)	60 (57.7%)	
menopause	46 (43%)	44 (42.3%)	
Tumor location (%)			0.98
upper outer quadrant	46 (43%)	45 (43.3%)	
lower outer quadrant	27 (25.2%)	28 (26.9%)	
lower inner quadrant	24 (22.4%)	21 (20.2%)	
upper inner quadrant	10 (9.3%)	10 (9.6%)	
Histological type (%)			0.52
ductal	97 (90.7%)	89 (85.6%)	
lobular	4 (3.7%)	6 (5.7%)	
others	6 (5.6%)	9 (8.7%)	
Estrogen receptor (%)			0.577
negative	25 (23.4%)	21 (20.2%)	
positive	82 (76.6%)	83 (79.8%)	
Progesterone receptor (%)			0.537
negative	34 (31.8%)	29 (27.9%)	
positive	73 (68.2%)	75 (72.1%)	
Ki-67 Group (%)			0.007
<14%	18 (16.8%)	34 (32.7%)	
≥14%	89 (83.2%)	70 (67.3%)	
HER2 (%)			0.046
negative	85 (79.4%)	93 (89.4%)	
positive	22 (20.6%)	11 (10.6%)	
Molecular subtypes (%)			0.126
luminal	83 (77.6%)	84 (80.8%)	
HER2 over expression	13 (12.1%)	5 (4.8%)	
TNBC	11 (10.3%)	15 (14.4%)	
Axillary ultrasound (%)			<0.001
suspicious	56 (52.3%)	10 (9.6%)	
unsuspicious	51 (47.7%)	94 (90.4%)	

ALN, axillary lymph node; HER2, human epidermal growth factor receptor 2; TNBC, triple-negative breast carcinoma.

### 3.2 Predictive performance of different DL models

#### 3.2.1 Pathological model

**Figure 2:**
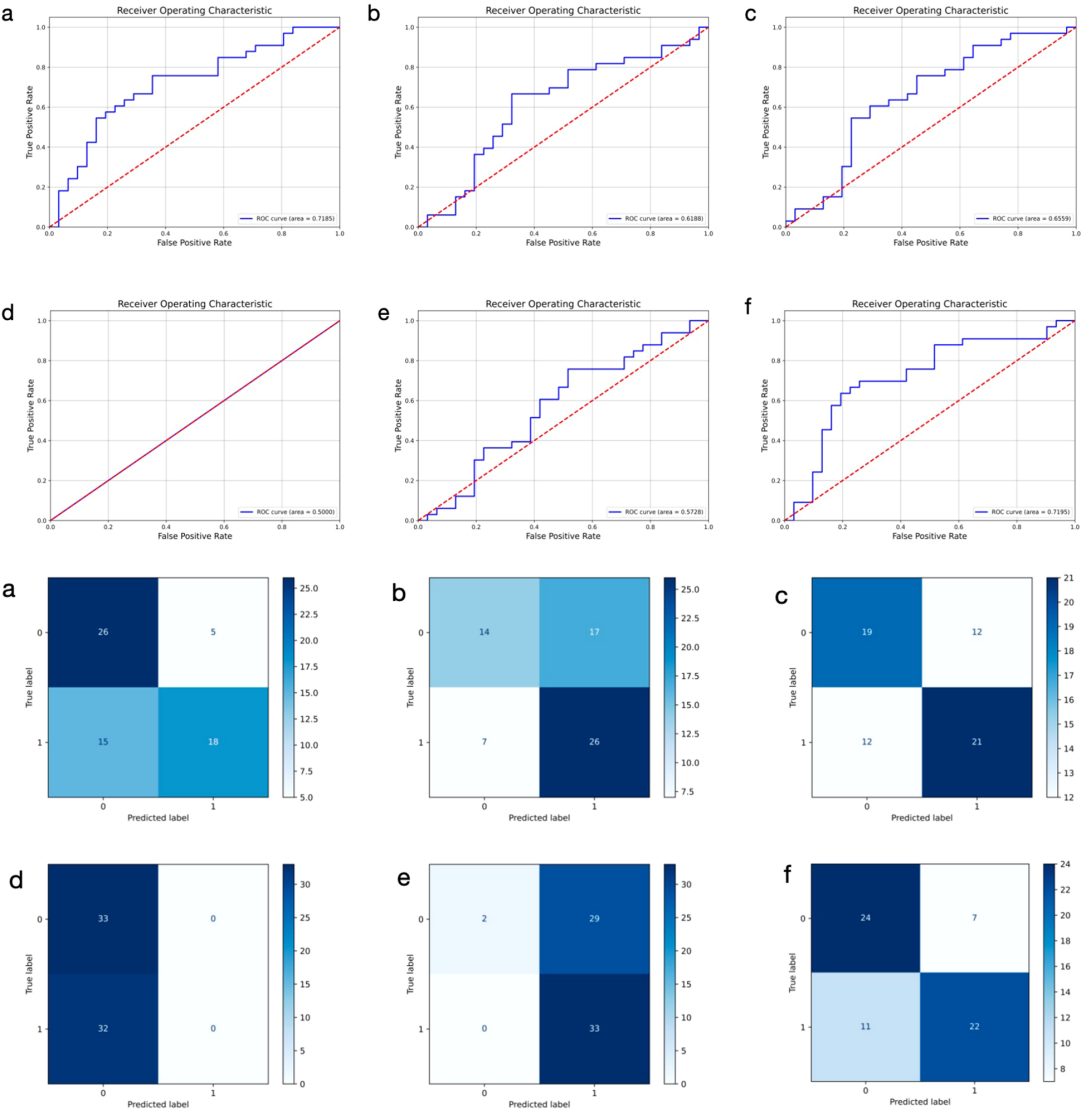
Receiver operating characteristic curves and confusion matrix of six different models using histopathological images for predicting axillary lymph node metastasis in breast cancer. 0: negative; 1: positive. **(a–f)** represent ResNet50, DenseNet, GoogLeNet, SqueezeNet, ViT, and PLNet model, respectively.

To assess the predictive performance of DL models using histopathological images of primary breast cancer, we evaluated several architectures, including ResNet50, DenseNet, GoogLeNet, SqueezeNet, ViT, and PLNet. The results, summarized in **Table 2**, indicate that PLNet exceeds the other five architectures across almost all metrics, except for a slightly lower recall value. The confusion matrices and ROC curves for all six models in predicting ALNM based on histopathological images are shown in **Figure 2**. Based on these findings, we selected PLNet as the primary DL architecture for histopathological images analysis in this study, with an AUC of 0.7195, accuracy of 0.7186, precision of 0.7188, recall of 0.6667, and F1-score of 0.7091.

**Table 2:** Predictive performance of deep learning models based on histopathological images for predicting ALN metastasis.

Method	Precision	Recall	F1-score	Accuracy	AUC
ResNet50	0.6875	0.5455	0.6429	0.7126	0.7185
DenseNet	0.625	0.7879	0.6842	0.6047	0.6188
GoogLeNet	0.625	0.6364	0.6364	0.6364	0.6559
SqueezeNet	0.5077	0	0	0	0.5000
ViT	0.5469	0.5734	0.6947	0.5323	0.5728
PLNet	**0.7188**	**0.6667**	**0.7097**	**0.7186**	**0.7195**

Bold values indicates the best result in deep learning models based on histopathological images. AUC, area under the curve.

#### 3.2.2 Imaging model

**Figure 3:**
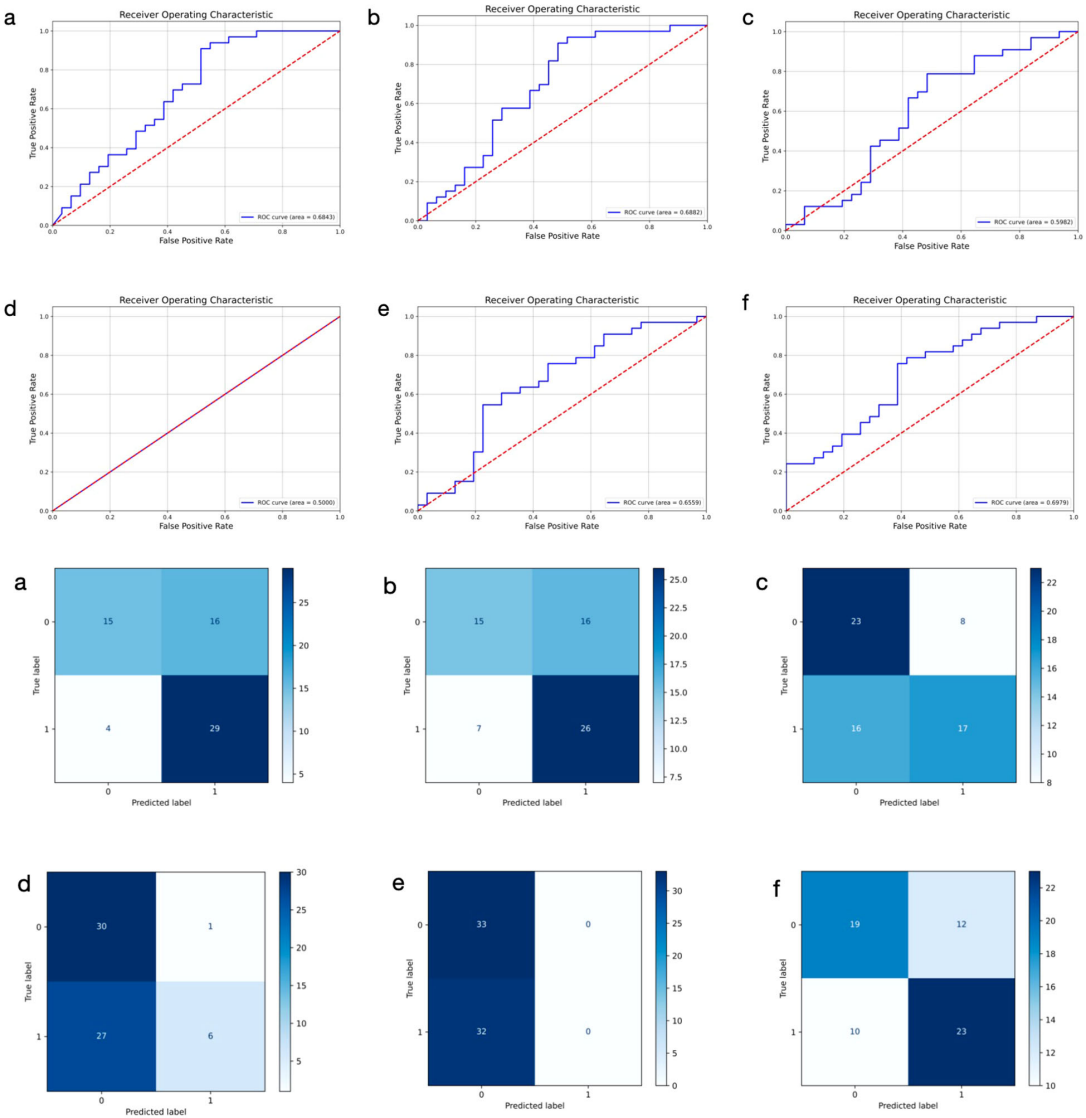
Receiver operating characteristic curves and confusion matrix of six different models using ultrasound images for predicting axillary lymph node metastasis in breast cancer. 0: negative; 1: positive. **(a–f)** represent ResNet50, DenseNet, GoogLeNet, SqueezeNet, ViT, and ULNet, respectively.

We trained six distinct models to extract DL features from primary breast cancer ultrasound images for the prediction of ALNM. As shown in **Table 3**, the ULNet model outperforms other models in terms of AUC (0.6979), precision (0.6875), recall (0.7488) and F1-score (0.7436). It achieved the best overall performance and was therefore selected as the primary DL architecture for ultrasound image analysis. Compared to the PLNet model based on histopathological images, the ULNet model, which is based on ultrasound images, demonstrated lower AUC, accuracy, and precision. However, the ULNet model improved the recall to 0.7488, demonstrating an improvement in recall after incorporating mask information. The confusion matrices and ROC curves for all six models are presented in **Figure 3**.

**Table 3:** Predictive performance of deep learning models based on ultrasound images for predicting ALN metastasis.

Method	Precision	Recall	F1-score	Accuracy	AUC
ResNet50	0.6562	0.697	0.6765	0.6571	0.6843
DenseNet	0.625	0.5152	0.5862	0.68	0.6882
GoogLeNet	0.6562	0.7276	0.6944	0.641	0.5982
SqueezeNet	0.5156	0	0	0	0.5000
ViT	0.5625	0.5471	0.541	0.6021	0.6559
ULNet	**0.6875**	**0.7488**	**0.7436**	**0.6444**	**0.6979**

Bold values indicates the best result in deep learning models based on ultrasound images. AUC, area under the curve.

It’s worth noting that the SqueezeNet model produced a recall and F1-score of 0 in both the ultrasound-based and pathology-based tasks, indicating that the model failed to correctly identify any breast cancer patients with ALNM. We attribute this failure to three interdependent factors: 1) Representational capacity deficit: The model’s extreme parameter compression (1.24M parameters *vs*. ResNet50’s 25.6M) critically limits its ability to capture histo-morphological discriminators of metastasis (e.g., micrometastases in capsule-distorted lymph nodes); 2) Feature space imbalance: Metastatic manifestations occupy sparse regions in the feature space, requiring specialized architectural components (e.g., attention mechanisms) absent in SqueezeNet; 3) Gradient dissipation: Vanishing gradients during backpropagation prevent effective weight updates. SqueezeNet, being an extremely lightweight architecture originally designed for resource-constrained environments, may lack sufficient capacity to capture the complex and high-dimensional patterns in medical imaging data-especially in the context of relatively subtle differences between metastatic and non-metastatic lymph node profiles. We intentionally chose to include SqueezeNet in our comparison to illustrate the limitations of overly simplified networks when applied to challenging clinical prediction tasks.

#### 3.2.3 Multimodal fusion model

**Figure 4:**
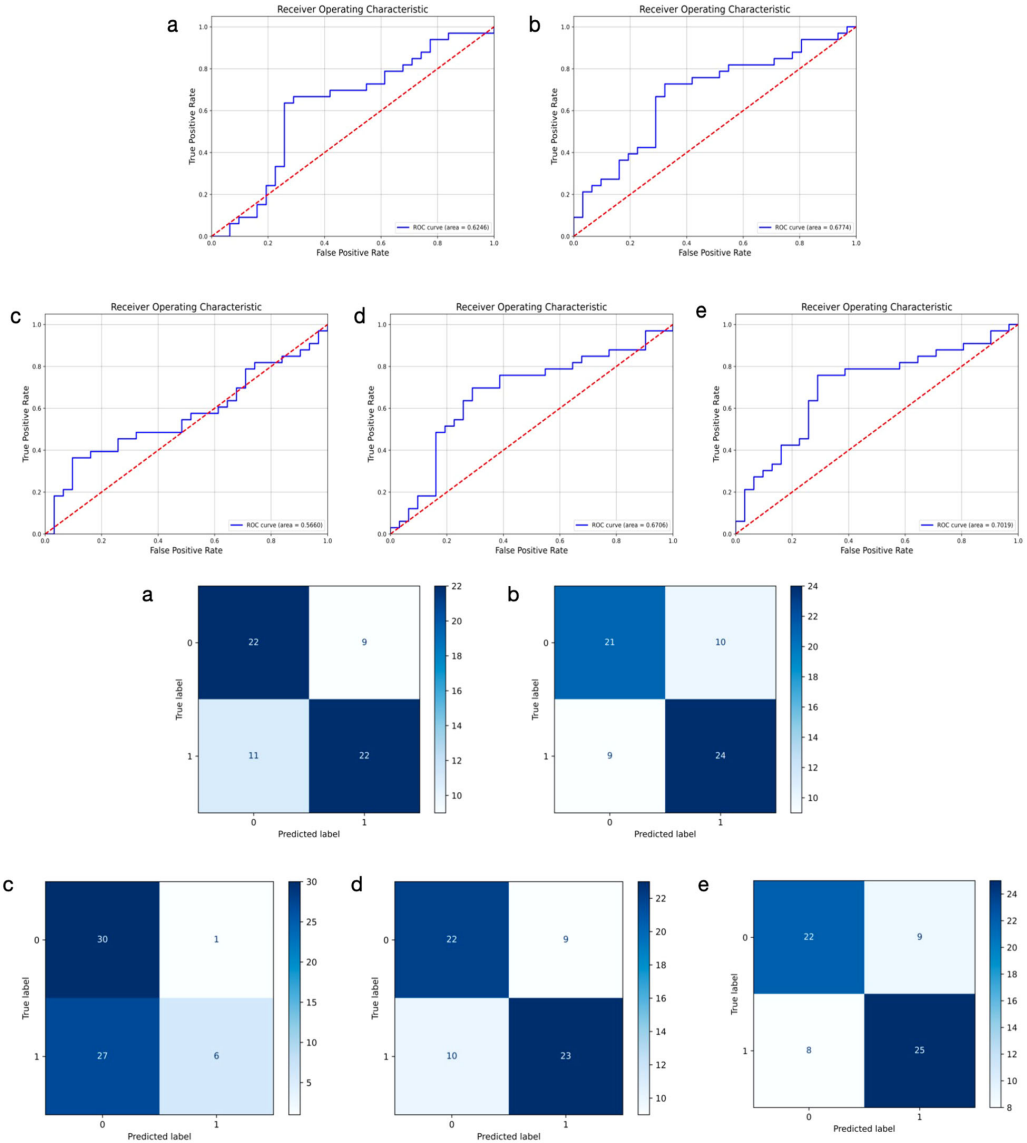
Receiver operating characteristic curves and confusion matrix of 5 different models using ultrasound and histopathological images for predicting axillary lymph node metastasis in breast cancer. 0: negative; 1: positive. **(a–e)** represent concatenation, weighting, attention, voting, and multilayer fusion, respectively.

We developed a multimodal approach that integrates features from both ultrasound and histopathological images of primary breast cancer to predict ALNM. The ULNet and PLNet were chosen as the base DL models for analyzing ultrasound and histopathological images, respectively. We experimented with multiple fusion strategies, including concatenation, weighting, attention, voting, and multilayer fusion. Among these, the multilayer-fusion model delivered the best performance, with an accuracy of 0.7353, precision of 0.7344, recall of 0.7576, and an F1-score of 0.7463 (**Table 4**). Performance was assessed using the ROC curve and a confusion matrix (**Figure 4**). Compared to single-modality models (based on either imaging or pathology), the multilayer-fusion model demonstrated an AUC of 0.7019, which was comparable to or slightly lower than that of the PLNet model (AUC = 0.7195). The combination of ultrasound and histopathological images does not improve the AUC, which may be due to factors such as architectural constraints or label noise. However, the multilayer-fusion model outperformed the single-modality models in terms of accuracy, precision, recall, and F1-score. when all performance metrics were considered, the multilayer-fusion model surpassed single-modality models based solely on ultrasound or histopathological images, demonstrating its effectiveness for predicting ALNM.

**Table 4:** Predictive performance of an integrated deep learning model based on ultrasound and histopathological images for predicting ALN metastasis.

Method	Precision	Recall	F1-score	Accuracy	AUC
concatenation	0.6875	0.6667	0.6875	0.7079	0.6246
weighting	0.7031	0.7273	0.7164	0.7059	0.6774
attention	0.5625	0.1818	0.3	0.5571	0.5660
voting	0.7031	0.697	0.7077	0.7188	0.6706
group1	0.7188	0.6667	0.7097	0.7186	0.7195
group2	0.6875	0.7488	0.7436	0.6444	0.6979
Multilayer-fusion	**0.7344**	**0.7576**	**0.7463**	**0.7353**	**0.7019**

Bold values indicates the best result in all deep learning models. AUC, area under the curve.

### 3.3 Modal visualization of ALNM prediction

We employed a heatmap visualization technique for the multilayer-fusion model to assess the confidence of its predictions, offering an intuitive view of model focus areas. The heatmap highlights regions activated by the model that are most indicative of lymph node metastasis, with blue and red areas representing regions that received higher attention and carry the greatest predictive significance. The deeper the color, the higher the likelihood of predicting ALNM.

**Figure 5:**
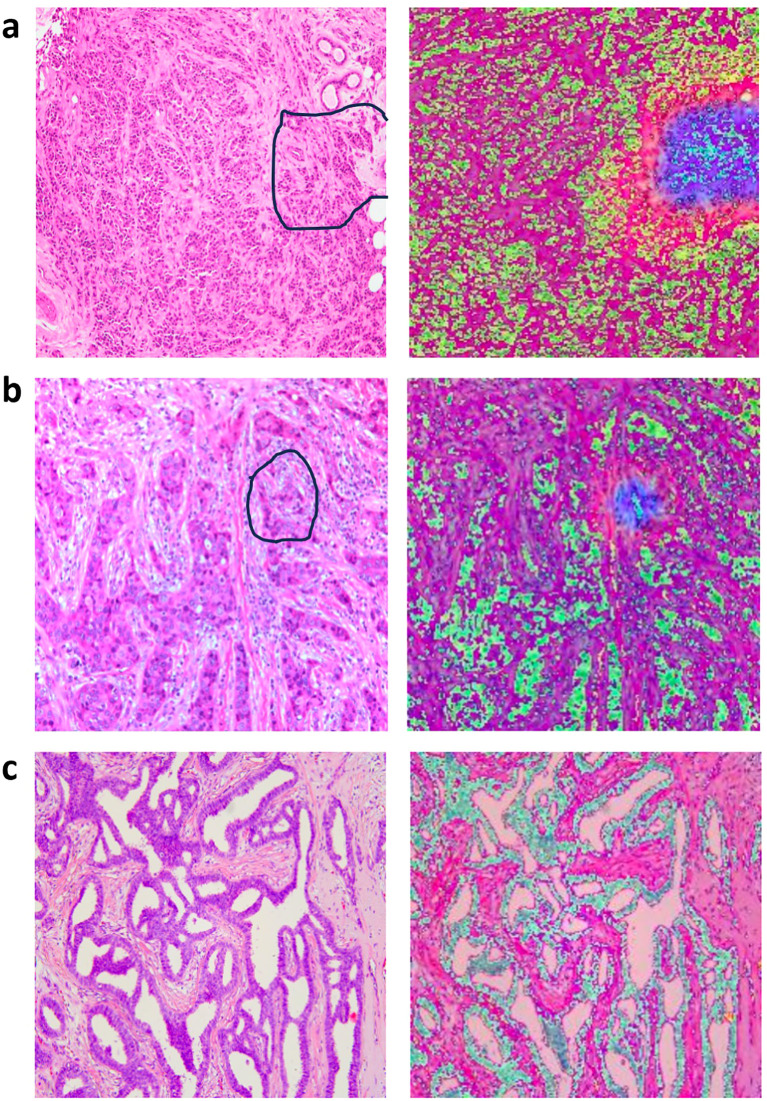
Representative original histopathological images and heatmaps. Histopathological images of primary breast with annotation markers (left column) and their corresponding attention heatmaps (right column), the darker the feature color, the higher the attention of the model. **(a)** The marked regions on the H&E slides represent the tumor-stroma interface, containing both apparently invasive carcinoma and abundant collagenous stroma (left column)**;** corresponding heatmaps are shown in the right column. **(b)** The marked regions on the H&E slides represents invasive carcinoma nests (left column), with their corresponding heatmaps shown in the right column. **(c)** The image in the left column represents H&E slides from a breast cancer patient without axillary lymph node metastasis, corresponding heatmaps are shown in the right column.

To pathologically validate our model’s attention mechanisms, two senior breast pathologists (>10 years specialty experience) conducted a blinded retrospective analysis of 50 randomly selected cases. Following standardized diagnostic protocols, they independently evaluated regions highlighted by the multilayer-fusion heatmaps against established histopathological criteria. High-attention areas were systematically assessed for correlation with clinically significant features including invasive carcinoma nests demonstrating aggressive morphological patterns (e.g., irregular infiltrative borders, desmoplastic stromal reactions) and dense collagenous matrix formations associated with metastatic propensity. The pathologists confirmed consistent spatial correspondence between model attention foci and these high-risk histological characteristics, particularly noting alignment in zones exhibiting lymphatic tumor emboli and peritumoral fibroblast proliferation. This qualitative concordance substantiates that our model prioritizes morphologically aggressive tumor phenotypes in its decision-making process. To illustrate these findings, we have incorporated representative pathological-thermal correlations with annotation markers in **Figure 5**, demonstrating the clinical relevance of attention mechanisms in identifying high-risk tumor regions.

## 4 Discussion

In recent years, DL has been applied to breast cancer diagnosis, risk stratification, prognosis prediction, and treatment response assessment (21–23). Common DL network architectures, including CNN, recurrent neural network, and ViT, have been explored for these tasks. Among them, ViT has demonstrated state-of-the-art performance on several image classification datasets (24). To address the insufficient diagnostic accuracy of single-modality imaging in cancer, researchers have developed DL-based multimodal fusion models that leverage multiple data types commonly found in medical records. For instance, Ishak Pacal et al. developed InceptionNeXt-Transformer, a novel hybrid DL architecture combining CNNs and ViTs for multi-modal breast cancer image analysis (25). A hybrid deep learning model that integrated InceptionNeXt blocks, enhanced Swin Transformer blocks, and a Residual Multi-Layer Perceptron for automated colorectal cancer detection (26). Additionally, the same team designed XtBrain, another novel hybrid architecture that combined local and global feature learning for brain tumor classification. XtBrain leverages the NeXt Convolutional Block and the NeXt Transformer Block synergistically to enhance feature learning (27). Suat Ince et al. proposed a novel U-Net architecture enhanced with ConvNeXtV2 blocks and GRN-based Multi-Layer for cerebral vascular occlusion segmentation (28).

In this study, we developed a multimodal fusion model integrating ultrasound and histopathology images to predict ALNM in breast cancer. HE-stained biopsy pathology images provide micro-level information on tumor tissue and cellular structures, though limited by sampling scope, while ultrasound captures macro-level tissue characteristics. The complementary integration of these data using a fusion model offers a more comprehensive view of tumor biology. Huang et al. reported an AUC of 0.900 (95% CI 0.819–0.953) for early breast cancer subtype differentiation using a fusion model of preoperative ultrasound and whole-slide images, highlighting the advantages of image fusion over single-modality models (29). Our results showed that, compared to single-modality models and other fusion strategies, the multilayer fusion model achieved better predictive performance (accuracy: 0.7353, precision: 0.7344, recall: 0.7576, F1-score: 0.7463, AUC: 0.7019). Our findings further validate the benefits of integrating preoperative ultrasound with biopsy pathology images. Although the multimodal fusion model achieved an AUC of 0.7019, indicating modest diagnostic performance. It serves as a complementary tool for guiding personalized treatment decisions. Future work is needed to optimize the model. Complementary to our multimodal imaging approach, Bove et al. demonstrated that combining clinical parameters with radiomic features from ultrasound images significantly improves nodal status prediction in clinically negative patients (18). While their methodology leverages handcrafted radiomic features and clinical integration, our deep learning-based fusion of raw ultrasound and histopathology images represents a distinct technical pathway toward the shared goal of refining axillary management decisions. Both studies underscore the critical value of multidimensional data integration in breast cancer staging.

Axillary lymph node involvement typically indicates a poorer prognosis and higher recurrence risk in breast cancer patients (8). Thus, preoperative accurate assessment of axillary lymph node status helps clinicians devise appropriate axillary treatment strategies, reducing postoperative complications and improving outcomes. Axillary ultrasound is commonly recommended as part of the standard diagnostic workup for patients with invasive breast cancer (30). However, its diagnostic accuracy is limited by high operator dependency. In our study, axillary ultrasound demonstrated a true positive rate of 52.3% and a false negative rate of 47.7%. This false negative rate is notably higher than that reported in previous studies, which range from 23.4% to 25% (31, 32), potentially leading to the underdiagnosis of patients with clinically significant nodal metastasis. Our multilayer fusion model presents an alternative approach for detecting ALNM in breast cancer patients, offering clinicians an alternative tool to inform treatment decisions. For patients without evidence of nodal involvement, axillary surgery could potentially be avoided, whereas sentinel lymph node dissection (SLND) or axillary lymph node dissection (ALND) is typically performed in cases with confirmed ALNM (33). Although ALND remains the gold standard for diagnosing axillary lymph node status and preventing axillary recurrence, it is associated with significant complications, including lymphedema, restricted mobility, and sensory abnormalities (33). Over the past few decades, axillary management has become less invasive, with SLND largely replacing ALND, thereby minimizing physical harm. The Z0011 trial demonstrated that for patients with one or two positive sentinel nodes, survival outcomes are comparable between those undergoing SLND alone and those undergoing ALND (34). As research increasingly emphasizes individualized and minimally invasive treatments (35, 36), it is crucial to accurately assess axillary lymph node status preoperatively, particularly in cases where clinical and imaging examinations show no suspicious signs of ALNM. SLND could be avoided if reliable preoperative evaluation of ALN status were available.

In our study, the deep learning model based on ultrasound achieved an AUC of 0.6979, while the histopathology-based model achieved an AUC of 0.7195, both of which are lower than those reported in previous studies (15, 17), likely due to the smaller sample size. In clinical practice, multi-modal imaging offers more complementary information compared to single-modal imaging. The multimodal fusion model integrating ultrasound and histopathological images achieved an AUC of 0.7019 - comparable to, or slightly lower than that of the histopathology-based model, suggesting no significant diagnostic improvement. However, in medical imaging, evaluating model performance based solely on the AUC value is insufficient. Other critical metrics, such as precision, accuracy, recall, and F1-score, must also be considered. When evaluating these comprehensive performance metrics, the multilayer fusion model outperformed all single-modality models in our study, demonstrating improvements in precision, accuracy, recall, and F1-score. Specifically, the multilayer fusion model showed superior precision and accuracy, indicating that its positive predictions were primarily true positives, and that it achieved a higher overall correct prediction rate, with fewer false positives and false negatives. In terms of recall, the multilayer fusion model also exhibited superior performance, identifying more true positive cases and thereby reducing the risk of missed diagnoses. Importantly, the F1-score, which balances precision and recall, was significantly higher for the multilayer fusion model, underscoring its advantage in comprehensively evaluating positive cases and minimizing missed diagnoses. These findings suggest that the multilayer-fusion model is proposed not solely based on AUC, but on its balanced and consistent advantage across multiple performance dimensions, demonstrating its comprehensive advantage in predicting ALNM. In our research, the multilayer-fusion model based on ultrasound and histopathological images does not improve the AUC, which may be due to factors such as architectural constraints or label noise. Future work will employ cross-modal attention mechanisms to better isolate complementary features across modalities. This could include incorporating more advanced models, such as EfficientNetV2 and Swin Transformer, to validate performance improvements (37).

In our multilayer fusion model strategy, a Squeeze-and-Excitation (SE) attention mechanism was incorporated to adaptively recalibrate feature channel weights, thereby enhancing the representation of critical features while suppressing irrelevant or noisy features. This mechanism increases the model’s sensitivity to key features, thus improving classification accuracy (38). After feature scaling, individual model features were concatenated, ensuring a more uniform feature value distribution and reducing instability during training due to large feature value discrepancies. To address significant feature differences across modalities, batch normalization (BN) layers were applied to both models before feature fusion. Given the substantial variation in the distributions of ultrasound and histopathology features, BN layers effectively standardize the features, making the fusion process smoother and more stable (39). After integration, the recall and accuracy of the fusion model were improved compared to the unimodal (imaging or pathological) models. The improvements in recall (likewise recognized as sensitivity) with our fusion model suggest it may minimize missed diagnoses of lymph node metastasis, proving particularly valuable for identifying high-risk patients. By providing reliable risk assessments, the fusion model can support clinical decisions on further invasive evaluation or personalized treatment planning, underscoring its potential as a clinical decision support tool.

To enhance the interpretability of our multilayer fusion model, we employed heatmap visualization to highlight the region’s most influential in the model’s predictions. For ultrasound images, the key regions for distinguishing ALNM in breast cancer were found to be the tumor boundary and the low-echo areas within the tumor. In histopathological slides, the regions exhibiting prominent invasive cancer cells and dense collagen stroma were identified as the most predictive features. These findings are consistent with previous studies that have linked ultrasonographic characteristics—such as tumor size, echogenicity, and lesion boundary—as well as histological features like grading and lymphatic vascular invasion, to axillary lymph node involvement in primary breast cancer (40–44). Additionally, our study highlighted that high Ki-67 expression and positive HER2 status may also serve as potential risk factors for ALNM, aligning with prior research (45–47). High Ki-67 expression, which reflects the proliferation activity of cancer cells, has been established as a significant predictor of ALNM (48, 49). Collectively, these findings further support the clinical reliability of our model’s predictions, providing a framework for predicting ALNM in breast cancer patients.

This study has some limitations. First, the small sample size increases the risk of overfitting and may affect the model’s generalizability. The model’s performance may not hold up in a larger, more diverse patient cohort. Future research should involve larger and more diverse cohorts to enhance the model’s robustness across different patient demographics, tumor types, and pathological stages. Second, selection bias is an inherent limitation of this retrospective research. The stability, generalizability, and clinical utility of the model requires further validation through prospective, multi-center studies. Additionally, while the multilayer fusion model outperformed other models, its specificity can be further improved to reduce false positives. Enhancing specificity may require more complex model structures or higher-resolution imaging, potentially achieving a better balance between sensitivity and precision.

## 5 Conclusion

Overall, this study developed a multimodal fusion model that integrates ultrasound images and pathology slides of the primary tumor to preoperatively predict ALNM in breast cancer patients. The model demonstrated a moderate diagnostic performance, outperforming models based solely on single-modality images. This approach has the potential to assist clinicians in lymph node staging and personalized treatment decisions. Future improvements could focus on enhancing predictive accuracy by incorporating larger datasets, leveraging more advanced architectures, and comprehensively integrating clinical features with multiple image data.

## Data Availability

The original contributions presented in the study are included in the article/supplementary material. Further inquiries can be directed to the corresponding authors.
